# Correction: A comprehensive review of enhanced CO_2_ capture using activated carbon derived from biomass feedstock

**DOI:** 10.1039/d6ra90030e

**Published:** 2026-03-26

**Authors:** Shreyase Kundu, Tasmina Khandaker, Md Al-Amin Mia Anik, Md. Kamrul Hasan, Palash Kumar Dhar, Sagar Kumar Dutta, M. Abdul Latif, Muhammad Sarwar Hossain

**Affiliations:** a Chemistry Discipline, Khulna University Khulna-9208 Bangladesh sarwar@chem.ku.ac.bd; b Department of Chemistry, Bangladesh Army University of Engineering & Technology (BAUET) Qadirabad Cantonment Natore-6431 Bangladesh; c Department of Chemistry, Begum Rokeya University Rangpur-5404 Bangladesh

## Abstract

Correction for ‘A comprehensive review of enhanced CO_2_ capture using activated carbon derived from biomass feedstock’ by Shreyase Kundu *et al.*, *RSC Adv.*, 2024, **14**, 29693–29736, https://doi.org/10.1039/D4RA04537H.

The authors regret that on page 29697, Section 2.2 paragraph 2, in the section beginning ‘Pecan nutshells were used as precursors…’ a reference was missed within the text.

The corrected section with appropriate reference are as included below.

Pecan nutshells were used as precursors for producing activated carbon through a one-step process. The nutshells were treated and mixed with two different chemical agents, potassium carbonate and potassium hydroxide, in a 1 : 1 ratio. Subsequently, this mixture was subjected to microwave pyrolysis at power levels of 300 W and 400 W for periods ranging from 2 to 6 min. Notably, microwave pyrolysis significantly improved the formation of microporous structures, especially in the samples activated with potassium hydroxide. For example, the samples pyrolyzed at 300 W for 6 min exhibited more than 73% ultra-microporosity. Additionally, these samples demonstrated an excellent CO_2_ adsorption performance, achieving 5.3 mmol g^−1^ at 1 bar and 0 °C. This level of performance was comparable to that of the activated carbon synthesized through conventional methods reported in the literature, underscoring the significant potential of microwave pyrolysis for future application in the production of activated carbon.^1^

1 G. D. Jiménez, L. A. Stevens, E. T. Kostas, V. H. Montoya, J. P. Robinson and E. R. Binner, Rapid, simple and sustainable synthesis of ultra-microporous carbons with high performance for CO2 uptake, *Chem. Eng. J.*, 2020, **388**, 124309, 10.1016/j.cej.2020.124309.

The Royal Society of Chemistry apologises for these errors and any consequent inconvenience to authors and readers.

